# Characterization of the Protein Corona of Three Chairside Hemoderivatives on Melt Electrowritten Polycaprolactone Scaffolds

**DOI:** 10.3390/ijms24076162

**Published:** 2023-03-24

**Authors:** T. Fernandez-Medina, C. Vaquette, M. N. Gomez-Cerezo, S. Ivanovski

**Affiliations:** 1 School of Dentistry, The University of Queensland, Brisbane 4006, Australia; 2 College of Medicine and Dentistry, James Cook University, Cairns Campus, Cairns 4870, Australia; 3 Departamento de Química en Ciencias Farmacéuticas, Facultad de Farmacia, Universidad Complutense de Madrid, Instituto de Investigación Sanitaria Hospital 12 de Octubre i+12, Plaza Ramón y Cajal s/n, 28040 Madrid, Spain

**Keywords:** polycaprolactone, hemoderivative, protein corona, regeneration

## Abstract

In tissue engineering, the relationship between a biomaterial surface and the host’s immune response during wound healing is crucial for tissue regeneration. Despite hemoderivative functionalization of biomaterials becoming a common tissue-engineering strategy for enhanced regeneration, the characteristics of the protein–biomaterial interface have not been fully elucidated. This study characterized the interface formed by the adsorbed proteins from various hemoderivatives with pristine and calcium phosphate (CaP)-coated polycaprolactone (PCL) melt electrowritten scaffolds. PCL scaffolds were fabricated by using melt electrospinning writing (MEW). Three hemoderivatives (pure platelet-rich plasma (P-PRP), leucocyte platelet-rich plasma (L-PRP) and injectable platelet-rich fibrin (i-PRF)) and total blood PLASMA (control) were prepared from ovine blood. Hemoderivatives were characterized via SEM/EDX, cross-linking assay, weight loss, pH and protein quantification. The interface between PCL/CaP and hemoderivative was examined via FTIR, XPS and electrophoresis. i-PRF/PCL-CaP (1653 cm^−1^), PLASMA/PCL-CaP (1652 cm^−1^) and i-PRF/PCL (1651 cm^−1^) demonstrated a strong signal at the Amide I region. PLASMA and i-PRF presented similar N1s spectra, with most of the nitrogen involved in N-C=O bonds (≈400 eV). i-PRF resulted in higher adsorption of low molecular weight (LMW) proteins at 60 min, while PLASMA exhibited the lowest adsorption. L-PRP and P-PRP had a similar pattern of protein adsorption. The characteristics of biomaterial interfaces can be customized, thus creating a specific hemoderivative-defined layer on the PCL surface. i-PRF demonstrated a predominant adsorption of LMW proteins. Further investigation of hemoderivative functionalized biomaterials is required to identify the differential protein corona composition, and the resultant immune response and regenerative capacity.

## 1. Introduction

In the context of tissue engineering, the relationship between a biomaterial surface and the host’s immune response during the early stages of wound healing is crucial for the subsequent regenerative outcome [1,2]. The early interaction of the biomaterial surface with the host’s immune cells, including neutrophils and monocyte/macrophages, is a key determinant of the subsequent inflammatory response that is triggered by the implanted biomaterial [3]. Ideally, the biomaterial should activate an inflammatory response that is resolved in a timely fashion, thereby facilitating the progression to tissue repair and regeneration. Conversely, a prolonged response (i.e., chronic inflammation) results in an adverse host response that leads to fibrous encapsulation and poor integration of the implanted biomaterial, also known as a foreign body response [4].

Immediately following surgical implantation of a biomaterial, the nature of the proteins that are adsorbed onto the implant surface is an important factor for the subsequent immune/inflammatory cell interaction [4]. Hence, an understanding of blood plasma adsorption, leading to the formation of a protein corona (PC) on the biomaterial surface, is essential for eliciting early wound-healing responses that subsequently lead to implant integration and/or tissue regeneration [5].

Extensively described for nanoparticles (NPs) and drug delivery [6], the PC refers to the double-layer of protein coating on a biomaterial interface [7]. The first layer, which is termed the “hard corona”, is strongly attached, whereas the outer layer, named the “soft corona”, is composed mainly of loosely adsorbed albumin protein and derivates [8]. According to the “Vroman effect” phenomenon, serum proteins, such as albumin, fibrinogen, fibronectin and other less abundant proteins [5,9], spontaneously adsorb and de-adsorb on biomaterial surfaces immediately post-implantation, thus forming the PC. The protein interaction between the hard and soft corona, as well as fibrinogen and platelets, defines the transitory matrix on a biomaterial surface [10,11,12,13]. The adsorbed plasma protein layer from the blood coagulation cascade interacts with blood cells that are chemoattracted to the site, thus determining the course of the early wound-healing stage [14,15,16]. Indeed, the plasma protein layer adsorbed on the biomaterial surface has been previously described [6,17,18] and is known to result in the chemoattraction of neutrophils and circulating monocytes [19,20]. Once the fibrin clot is established, the recruitment of innate immune cells rapidly triggers the inflammatory response [21,22]. Various studies have reported that the physicochemical properties of biomaterials and the nature of the biological environment can influence the distribution and composition of the PC and its downstream effect on the immune response [23,24,25]. 

The “hard corona” includes hundreds of different proteins that interact with the biomaterial surface in a dynamic and time-dependent manner, resulting in a continually changing PC composition, as previously described for polystyrene NPs [26]. Characterization of protein adsorption on NPs manufactured from polyesters, such as poly (lactic-co-glycolic acid) (PLGA) and polycaprolactone (PCL), has been reported [27] and demonstrated that albumin, Apolipoprotein A-I, Apolipoprotein B-100, serotransferrin and Complement C3 are the most abundant adsorbed plasma proteins. The adsorption of these proteins on hydrophobic surfaces (such as PCL) is mediated by the presence of lipid domains within these protein complexes [28,29,30]. 

The composition of the PC and its temporal evolution has been associated with various immune responses after biomaterial implantation [31]. The innate immune response can be modified by using several techniques that change either the biomaterial surface topography or chemistry (via the addition of functional groups), or both, although the precise biological mechanisms remain elusive [31]. The PC and the biophysical interaction with its functional groups have been demonstrated to exert a crucial influence on the host’s response to the implantation of a foreign body [4,32]. Complement component C3b, one of the most abundant lipoproteins in blood plasma, has been shown to covalently link to -OH groups, resulting in the activation of the inflammatory receptor Mac-1 on CD11b^+^ cells (neutrophils and macrophages), as an example. This serves as a damage-associated molecular pattern (DAMP) receptor that influences various immune cell responses including cytotoxicity, cellular activation and chemotaxis [33,34]. Surface chemistry can also affect the conformation of adsorbed proteins on biomaterials and, hence, regulate the immune cellular response [35,36]. This illustrates the importance of understanding the PC formed on biomedical devices to predict the subsequent immune response. Indeed, surface modification has been a key strategy for enhancing biomaterial bioactivity by orchestrating the early post-implantation inflammatory response [37] toward enhanced tissue regeneration.

An emerging strategy for improving the regenerative performance in biomaterials, especially bioinert polymers such as PCL, is their functionalization with different hemoderivatives, such as platelet-rich plasma (PRP) and fibrin (PRF) [38]. Indeed, PRP has been widely and empirically applied in soft and hard tissue reconstruction with the aim of accelerating and improving healing [39]. These blood products are obtained by the fractionation of human blood after centrifugation, creating a phase separation gradient between the protein liquid plasma and the cellular component. Growth factors, such as vascular endothelial growth factor (VEGF), fibroblast growth factor (FGF) and insulin-like growth factor-1 (IGF-I), are present at higher concentrations in hemoderivatives, including injectable platelet-rich fibrin (i-PRF), pure platelet-rich plasma (P-PRP) and leukocyte platelet-rich plasma (L-PRP), as compared to blood [40]. However, while the combination of hemoderivatives and biomaterials has become popular, the impact of the differential hemoderivative protein content on PC formation has not been well elucidated [41]. To address this, the present study characterized the interface formed by the protein adsorption from three different hemoderivatives (i-PRF, P-PRP, L-PRP) on pristine and calcium phosphate (CaP)-coated PCL melt electrowritten scaffolds. 

## 2. Results

### 2.1. SEM Characterization

**Figure 1:**
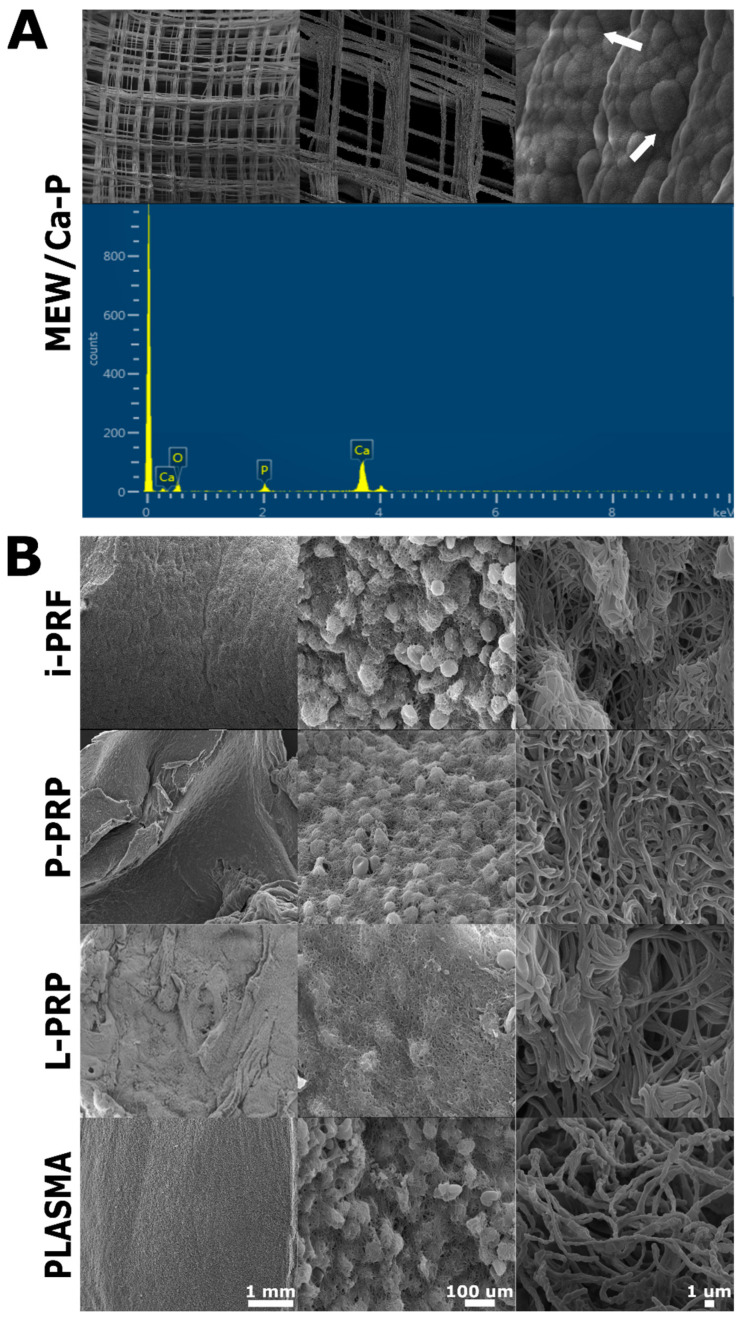
(**A**) SEM images from MEW scaffolds showing CaP surface deposition (far right image, white arrows) confirmed by energy-dispersive X-ray spectroscopy (EDX), as previously reported by our group [42]. (**B**) Surface characteristics of the different hemoderivatives including PLASMA.

SEM characterization was conducted for the scaffolds coated with the three hemoderivative and PLASMA groups. Imaging confirmed the presence of the CaP coating on the PCL surface of bare (without hemoderivative) melt electrospinning writing (MEW) PCL/CaP scaffolds images (Figure 1). Further EDX analysis confirmed Ca-P deposition on the PCL surface (Figure 1A). Characteristics of PCL/CaP scaffolds supplemented with hemoderivatives are provided in the Supplementary Material (Figure S1).

### 2.2. Blood Product Characterization

**Figure 2:**
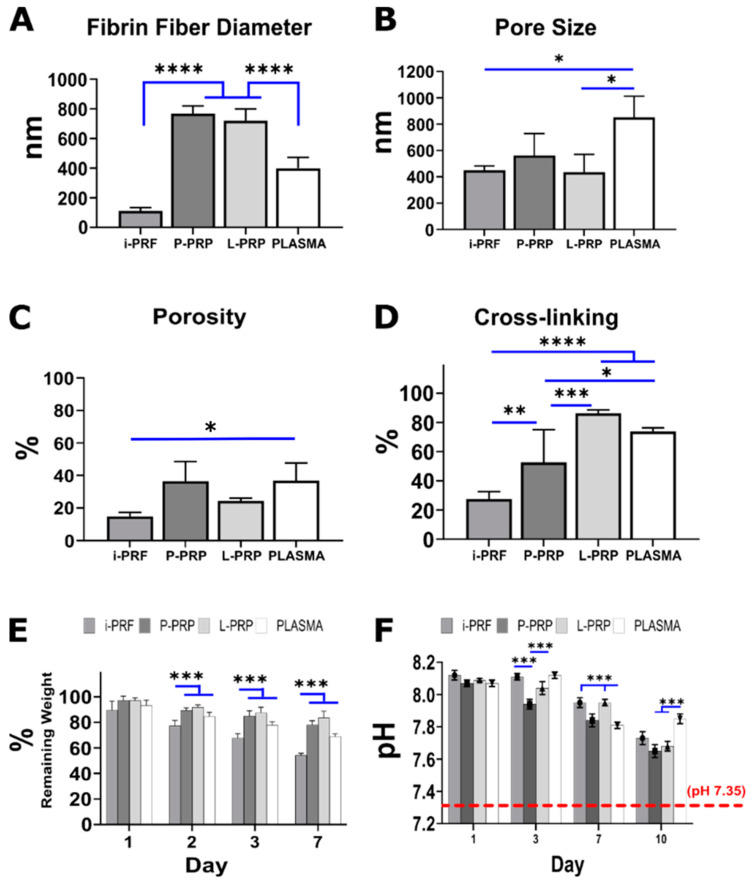
Characterization of the fibrin network within the different hemoderivatives. Fibrin fiber diameter (**A**), pore size (**B**) and porosity (**C**) were determined via SEM images. Cross-linking (**D**), weight loss (**E**) and pH (**F**) using media calibrated a pH 7.35 physiological value (red dotted line) as reference. Results are expressed in mean and standard deviation. Statistically difference was determined at (**** *p* < 0.0001), (*** *p* < 0.001), (** *p* < 0.01) and (* *p* < 0.05).

Varying physical properties, such as fibrin fiber diameter, pore size, % porosity, cross-linking, weight loss and pH, were observed for the different hemoderivative groups. Quantification of fibrin diameter (Figure 2A) revealed that P-PRP (767.1 ± 51.9 nm) and L-LRP (720 ± 78 nm) had significantly thicker fibrin fibers compared to PLASMA (398.7 ± 73.1 nm) and i-PRF (111.3 ± 21.4 nm). There was no statistically significant difference between P-PRP and L-PRP. Upon evaluation of the fibrin network pore size, PLASMA demonstrated the largest value and was significantly greater than L-PRP and i-PRF (* *p* < 0.05) (Figure 2B). There was a statistically significant difference between i-PRF and PLASMA (* *p* < 0.05) in % porosity (Figure 2C). Cross-linking efficacy (Figure 2D), which was assessed via a Ninhydrin assay, was lowest for i-PRF (27.4 ± 5.1%) and was significantly smaller when compared to the other blood products. L-PRP had the highest cross-linking ratio of 86.2 ± 2.3%, closely followed by PLASMA (73.8 ± 2.5%), with both groups being significantly greater than P-PRP (52.7 ± 22.3%).

The lower percentage of cross-linking was associated with increased weight loss (Figure 2E) when immersed in medium. Indeed, i-PRF displayed statistically higher weight loss when compared to the other blood products (except at the 1-day time point, where the difference was not statistically significant). All blood products demonstrated a gradual decrease in the percentage of remaining weight, although the L-PRP and P-PRP were the most stable, exhibiting approximately 20% weight loss at 7 days, as opposed to 30% and 50% weight loss for PLASMA and i-PRF, respectively. The pH values (Figure 2F) were measured at 1, 3, 7 and 10 days and revealed that all the blood products created a slightly alkaline environment with a pH of approximately 8, which gradually decreased toward physiological pH after 10 days of immersion. pH physiological value (pH ≈ 7.35) is shown as a reference (Figure 2F). The differences between P-PRP vs. i-PRF and vs. PLASMA were statistically significant (*** *p* < 0.001) at 3 days. Statistically significant differences for PLASMA vs. i-PRF and PLASMA vs. L-PRP were found at day 7 (*** *p* < 0.001). A similar trend was observed at day 10 showing differences between PLASMA vs. P-PRP and L-PRP (*** *p* < 0.001). However, all hemoderivatives demonstrated significant differences (*** *p* < 0.001) when compared to the PLASMA (control) group (pH 7.35) at all time-points.

### 2.3. Protein Absorption from Hemoderivatives

**Figure 3:**
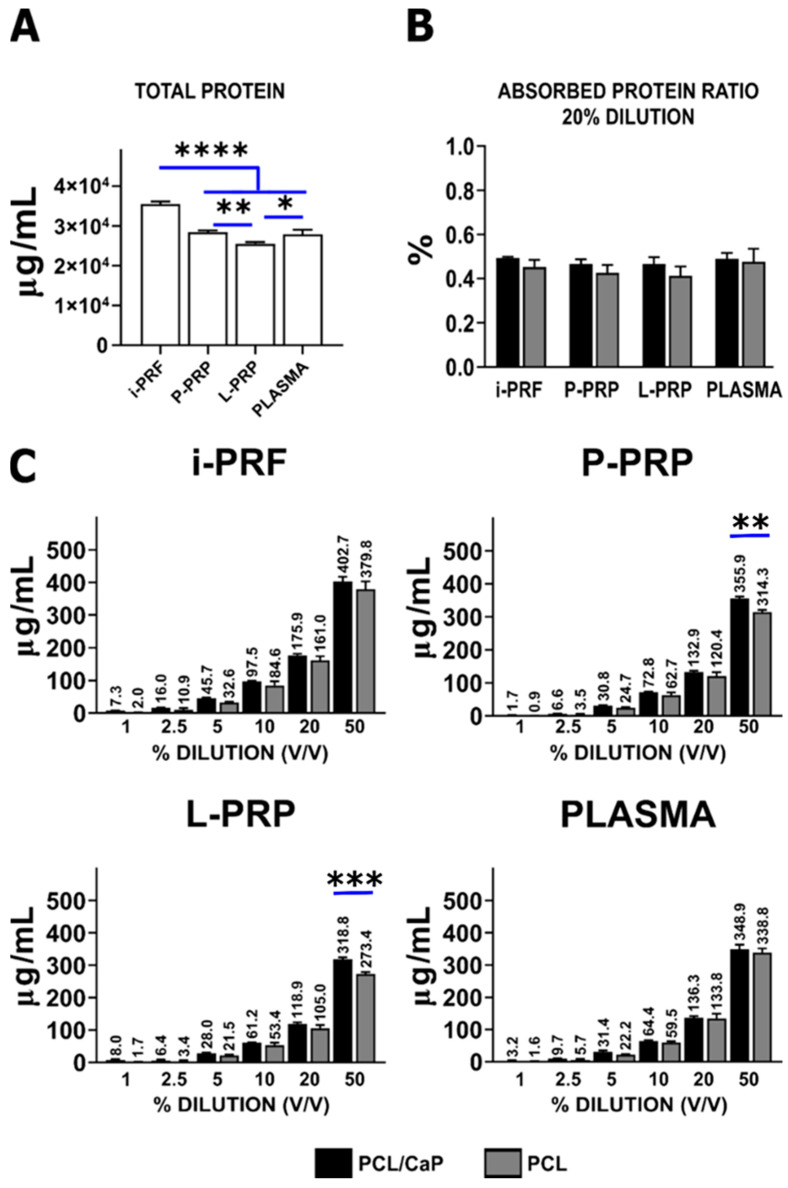
(**A**) Total protein quantification for each hemoderivative. (**B**) At 20% dilution, no statistically significant differences were found between the hemoderivatives applied on CaP scaffolds. (**C**) Hemoderivative protein adsorption at different dilutions showing statistically significant starts over time with increasing dilution in P-PRP and L-PRP. Other statistical significance marked by asterisks (**** *p* < 0.0001), (*** *p* < 0.001), (** *p* < 0.01) and (* *p* < 0.05).

Protein adsorption on the pristine PCL and PCL/CaP surfaces was evaluated via BCA assay by using different concentrations of the hemoderivatives. Total protein content associated with the various hemoderivatives was higher for i-PRF (35,510 ± 663.6 µg/mL) compared to P-PRP (28,466.7 ± 415.3 µg/mL), PLASMA (27,916.7 ± 1129.7 µg/mL) and L-PRP (25,500 ± 452.1 µg/mL) (**** *p* < 0.0001) (Figure 3A).

The effect of CaP coating on protein adsorption using a 20% of dilution (Figure 3B) has demonstrated comparable results across the groups, independently of the CaP coating. This dilution was individually evaluated by using the absorbed protein ratio, as it has been used to test anabolic effects and the cellular bioactivity of various hemoderivatives [40]. 

Total surface protein adsorption on PCL/CaP and PCL was evaluated for various hemoderivative dilutions. An exponential increase of protein adsorption was observed for all the hemoderivatives for concentrations of 1%, 2.5%, 5%, 10%, 20% and 50%. Although the CaP-coated scaffolds displayed slightly enhanced protein adsorption, this did not reach statistically significant differences for any of the hemoderivatives (Figure 3C). At 50% dilution, statistical difference was observed for P-PRP (355.9 ± 5.2 µg/mL) (314.37 ± 6.6 µg/mL) (** *p* < 0.01), and L-PRP (318.7 ± 5.6 µg/mL) (273.4 ± 6 µg/mL) (*** *p* < 0.001) only. 

### 2.4. Fourier-Transform Infrared Spectroscopy (FTIR)

**Figure 4:**
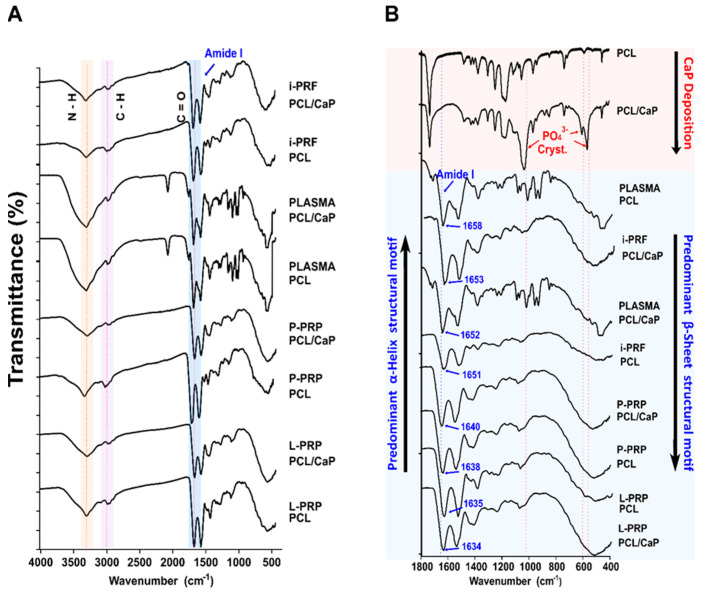
FTIR spectra for the various hemoderivatives and CaP scaffold combination. (**A**) All peaks in the N-H stretching region are significant above 3400 cm^−1^, especially on PLASMA/PCL/CaP. (**B**) Magnification of the stretching region encompassing 1800–400 cm^−1^ confirmed apatite deposition on PCL (1028, 600 and 561 picks) and the predominant α-Helix structural motif for i-PRF and predominant β-sheet motif for the anticoagulated protocols (i.e., P-PRP, L-PRP observed in the Amide I region). Colored bands represent Amide A (orange/N-H) or B (purple/C-H) from 3300 and 2900 cm^−1^ respectively, as well as Amide I (blue/ C=O) which appear at lower wavelengths from 1650 cm^−1^.

FTIR spectra were utilized to investigate potential conformational changes of the adsorbed hemoderivative proteins on both pristine PCL and PCL/CaP melt electrowritten scaffolds. Firstly, the presence of the CaP layer on the PCL surface was confirmed by the characteristic peaks of phosphate (PO43-) at 1028 cm^−1^, 600 cm^−1^ and 561 cm^−1^. Then, the Amide A and Amide I spectral signatures, at 3400 cm^−1^ and 1645 cm^−1^, respectively, were investigated. The analysis showed that conformation of the Amide I and Amide A regions was not observed when PLASMA was adsorbed onto the pristine PCL scaffold (Figure 4A). However, analysis of the structural conformation of Amide I between the wavelengths of 1800 cm^−1^ and 400 cm^−1^ (Figure 4B) revealed a minor formation of Amide I in the PLASMA/PCL group at 1658 cm^−1^, which was characteristically observed during protein deposition on surfaces.

Furthermore, i-PRF/PCL/CaP (1653 cm^−1^), PLASMA/PCL/CaP (1652 cm^−1^) and i-PRF/PCL (1651 cm^−1^) demonstrated a strong signal at the Amide I region that is predominantly associated with an α-Helix structural motif [43]. On the other hand, P-PRP/PCL/CaP with an Amide I peak at 1640 cm^−1^, P-PRP/PCL at 1638 cm^−1^, L-PRP/PCL at 1635 cm^−1^ and L-PRP/PCL/CaP at 1634 cm^−1^ all demonstrated a consistent tendency to display a predominant β-sheet related structure.

These results suggest that the presence of a hydrophilic CaP coating on the surface of the PCL plays only a minor role on determining the nature of the adsorbed protein conformations. However, the type of hemoderivative (and its associated specific protein content and composition) appears to be the determining factor for protein confirmation, as those preparations that underwent sequential centrifugation (P-PRP and L-PRP) displayed a β-sheet-related structure, whereas those with a composition closer to that of blood (i-PRF and PLASMA) resulted in the formation of an α-Helix. 

### 2.5. XPS Spectra

XPS evaluation was performed to further evaluate the protein composition of each hemoderivative fraction adsorbed on the PCL/CaP scaffolds only. Table 1 shows a summary of the C, O and N proportions as determined by XPS, confirming the adhesion of the proteins on the scaffolds surface. In addition, the O/C and N/C ratios were calculated to evaluate the composition of each hemoderivative group. O/C ratio was similar for i-PRF, P-PRP and PLASMA but was lower in L-PRP. The N/C ratio also showed differences in the relative quantity of nitrogen, with the plasma fraction possessing the highest relative concentration. 

**Figure 5:**
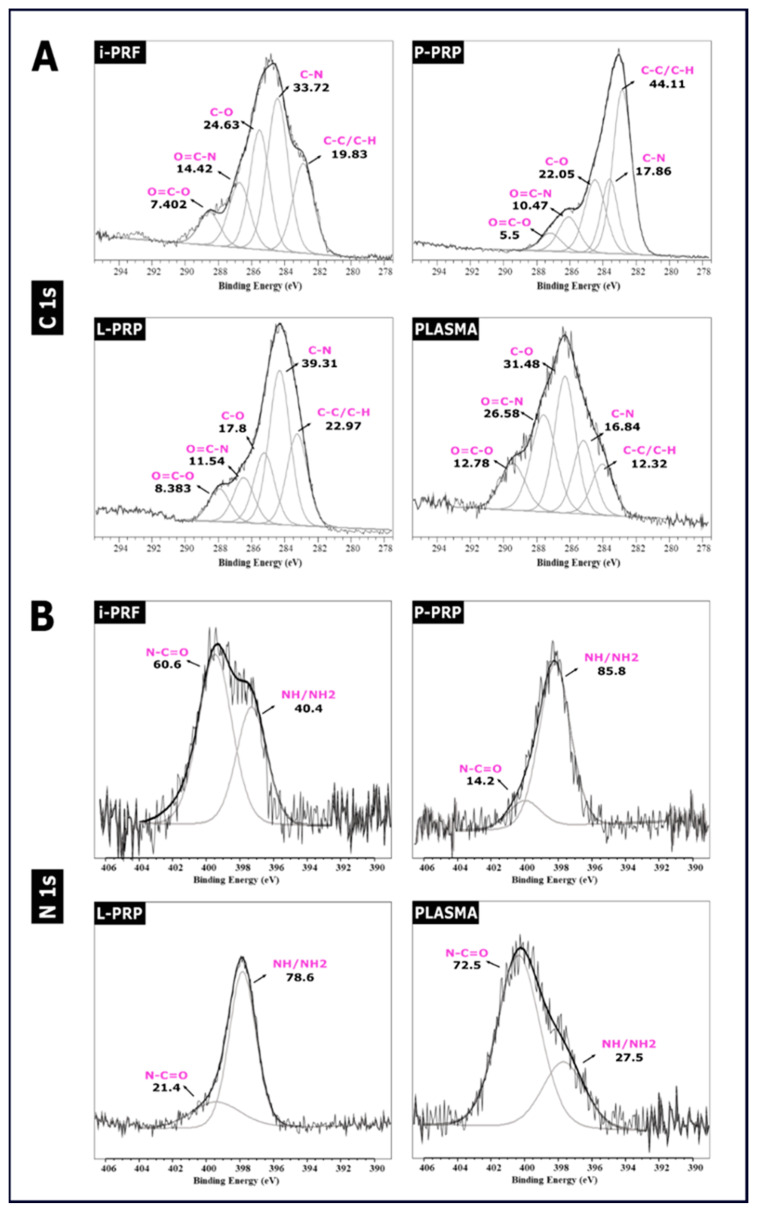
(**A**) C1s narrow scan and (**B**) N1s narrow scan XPS spectra for the different hemoderivatives on the PCL/CaP scaffold surface. Peak deconvolution of C1s and N1s of the different groups.

Figure 5A shows curve fitting of the high-resolution XPS spectra of carbon (C1s) and nitrogen (N1s), which are commonly used parameters to evaluate protein deposition on different surfaces [44,45]. PLASMA and i-PRF demonstrated a higher content of C-N (≈284 eV) and C-O (≈285 eV), whereas P-PRP and L-PRP exhibited higher quantities of C-C/C-H (≈283 eV). The N1s narrow spectra for all groups demonstrate differences in the nitrogen composition between the samples (Figure 5B). Consistent with C1s, PLASMA and i-PRF presented similar N1s spectra, with the majority of the nitrogen involved in N-C=O bonds (≈400 eV), while N-H/N-H2 bonds (≈397 eV) were lower at 40.4%, as previously reported [46]. 

P-PRP and L-PRP presented a completely different pattern, with most of the nitrogen involved in N-H/N-H2 bonds and only about 15–20% involved in N-C=O bonds. Moreover, the amount of NH2 was quantified by using the XPS, and this demonstrated that P-PRP and L-PRP had the highest values compared to the other groups. As will be further explored in the next section, these data show the same pattern as that observed with the NH2 quantification of cross-linking.

### 2.6. Protein Dynamic Binding

The protein deposition from the different hemoderivatives on the PCL/CaP surface was evaluated via BCA and SD-PAGE assays after exposure for 1, 5, 15, 30 and 60 min. This protein dynamic binding allows the evaluation of protein composition via molecular mass (kDa) as a function of time (min) and, hence, provides information of the protein adsorption–desorption patterns occurring on the biomaterial surface.

**Figure 6:**
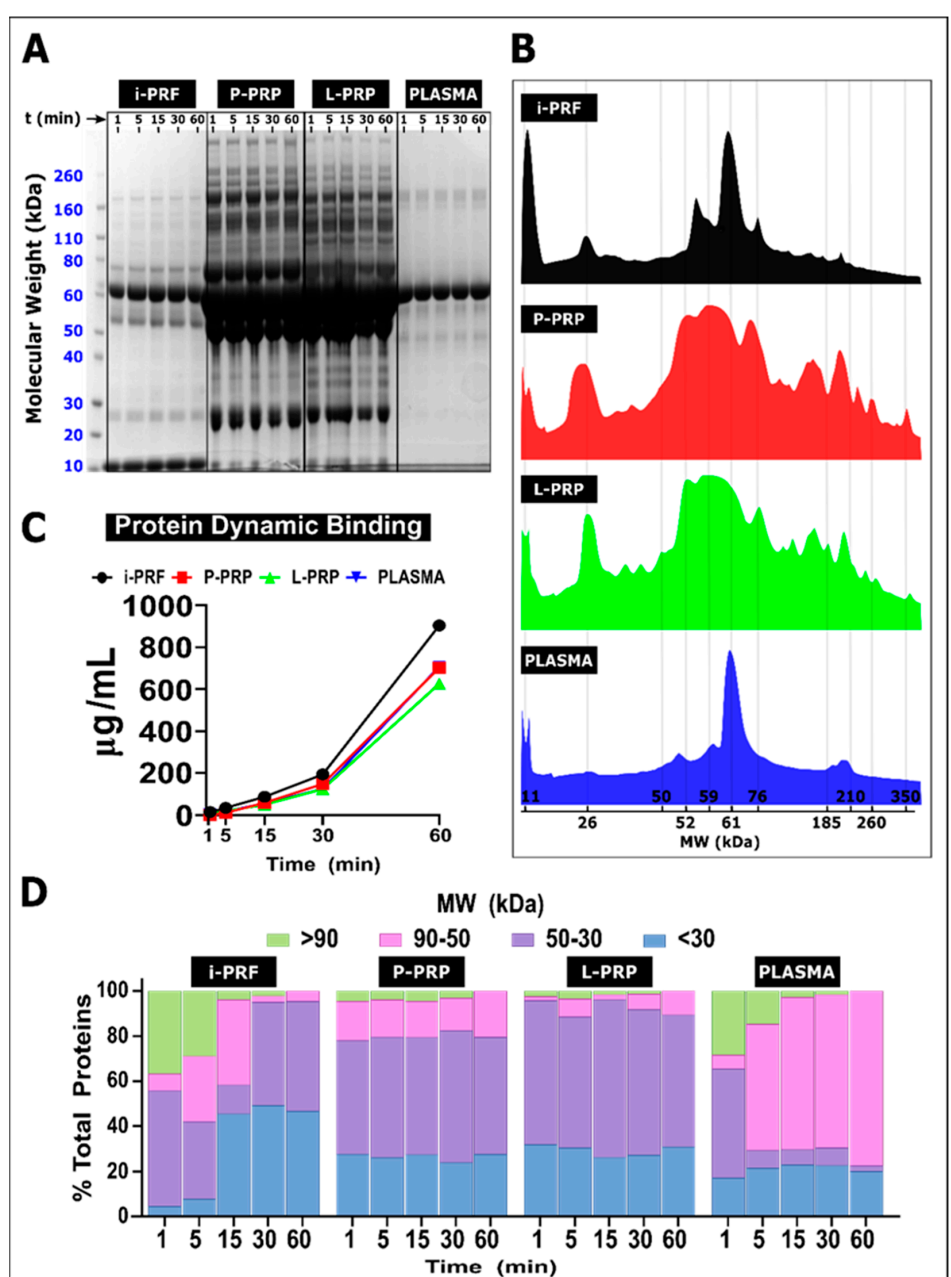
PC characterization. (**A**) SD–PAGE gel of the different groups at the 5 time points. (**B**) Spectrum of the protein intensity at 60 min segmented by their molecular weight (MW), showing the differential PC behavior analyzed by the area under the curve. (**C**) Total protein dynamic binding of different groups. (**D**) The protein deposition on the surface demonstrated differential protein composition by MW distribution on the PCL/CaP surface interface over time. At the early stages, predominantly high MW (>90 kDa) protein deposition was observed, followed by a secondary protein binding with medium and low MW (>30 kDa).

An overview of the protein adsorption on the biomaterial surface, as demonstrated via SDS-PAGE at time points ranging from 1 to 60 min, is shown in Figure 6A. Strong deposition of proteins between 50 and 65 kDa is evident for all groups, especially P-PRP and L-PRP. Moreover, similarities were also observed between i-PRF and PLASMA. A representation of the PC cumulative spectrum signature is provided by the relative intensity of the individual bands plotted against protein MW after 60 min of immersion in the various blood products (Figure 6B). This revealed that L-PRP and P-PRP presented a similar pattern of MW distribution, with the presence of 4 main peaks at 11, 26, 50–75 (broad peak) and 160–210 kDa (dual peak), suggesting that a similar protein composition was adsorbed on the PCL/CaP with these two hemoderivatives. PLASMA and i-PRF also presented some level of similarities in the PC signature with the presence of a peak at 11 kDa and a much narrower peak at 61 kDa when compared to L-PRP and P-PRP, further confirming the differential nature of the resulting PC for these blood products. Total protein dynamic binding (Figure 6C) revealed a similar amount of protein deposition for all groups at the early time points (1, 5, 15 and 30 min). However, i-PRF resulted in higher protein adsorption at 60 min, while PLASMA has the lowest value, and L-PRP and P-PRP again had an equivalent amount of protein adsorption. 

Furthermore, the percentage of four different protein groups categorized according to MW (high (>90 kDa), middle–high (90–50 kDa), middle–low (50–30 kDa) and low (<30 kDa)) was plotted against time to provide a visualization of the adsorption–desorption events occurring at the biomaterial surface for the various hemoderivatives (Figure 6D). After 1 min, i-PRF and PLASMA displayed a similar protein deposition profile with approximately 30% of proteins having high MW (≈35%), 10% middle–high MW, a predominant fraction of middle–low MW proteins (≈50%) and 5–10% of proteins with a low MW. These early timepoint similarities disappeared at subsequent time points as the high MW fraction was gradually replaced by low and middle–low MW for the i-PRF group (reaching 50 and 45%, respectively, at 60 min), whereas the fraction of middle–high MW protein became more evident in the PLASMA group, reaching 80% of the proteins.

Interestingly, P-PRP and L-PRP demonstrated similar patterns of protein MW distribution at almost all time points, with ≈25% for low MW and ≈50% for middle–low MW, and while the middle–high MW fraction was higher in the P-PRP (≈20%) compared to the L-PRP (≈5%) group, the high MW fraction was virtually non-existent. This MW distribution was relatively stable throughout the time of immersion, suggesting a rapid stabilization of the PC for these hemoderivatives. 

In summary, this experiment demonstrated that the chairside manufacturing of hemoderivatives is a potent and facile manner of modifying PC on PCL/CaP fibers and that each of these blood products has a distinct protein signature. 

## 3. Discussion

In this study, the protein adsorption on the biomaterial surface associated with various hemoderivatives was characterized on melt electrowritten PCL scaffolds. By using a simple chairside method of total blood peripheral centrifugation, it was demonstrated that it is possible to create a different PC composition on the biomaterial surface, which can potentially influence the subsequent cellular events leading to either regeneration or fibrous encapsulation. The control over both protein adsorption and the immune response triggered by the biomaterials is generally achieved by altering its surface chemistry or topography [47]. The data shown here suggest that pre-immersion in a hemoderivative is likely to significantly enhance bioactivity, especially in combination with surface functionalization treatments such as CaP. Previous reports have shown that the content of these hemoderivatives consists of highly abundant plasma proteins, such as Alpha-1-acid glycoprotein 2, Thrombospondin-1 (Glycoprotein G), albumin and Apolipoprotein A-I [48,49], as well as the common growth factors VEGF, PDGF-BB and BMP-2 [41,50]. L-PRP, P-PRP and i-PRF have many similarities, although the ratio of these constituent proteins differs. This may account for the unique PC formed by the proteins adsorbed from these blood preparations, potentially leading to a different immune response and downstream wound healing. 

FTIR revealed that the type of hemoderivative also affected the predominant protein conformation on the biomaterial surface [51,52]; protein adsorption from i-PRF resulted in the formation of a predominantly α-helix structural motif in the Amide I region (≈1650 cm^−1^), which has previously been associated with the presence of transmembrane proteins involved in the enhancement of cellular bioactivity [53]. In contrast, citrated blood products (L-PRP and P-PRP) displayed a predominant β-sheet structural motif in the Amide I region, which has been linked to fibrotic response and amyloidosis [54,55]. In addition, the predominant formation of the β-sheet has been reported to favor hemoderivative cross-linking [56], which is consistent with the findings of this study that the PRPs resulted in higher fibrin reticulation.

The composition, conformation and adsorption kinetics of adsorbed protein were strongly dependent on the type of hemoderivative that was used. This complex and dynamic interaction between proteins and the biomaterial surface is consistent with previous reports, albeit by using total blood plasma only as a biological fluid model [26,57,58,59]. Here, the development of the PC from the different hemoderivatives was analyzed via electrophoresis to further explore the dynamics of protein binding to the biomaterial surface. In agreement with previous reports, albeit by using nanoparticles, the development of the PC was characterized by rapid protein adsorption on the PCL/CaP surface regardless of the hemoderivative [26,60]. A substantial deposition of proteins with MW ranging from 46 kDa to 65 kDa was observed predominantly for L-PRP and P-PRP. This MW range has been previously attributed to the γ- and β-chains of fibrinogen that upregulate the inflammatory NF-κB pathway via integrin activation of leukocytes leading to the release of inflammatory cytokines [61,62]. In contrast, the use of i-PRF resulted in a major fraction of adsorbed proteins with a lower MW (below 30 kDa), including an intense peak at 10 kDa. This latter region has previously been linked to immunomodulatory cytokines, such as CCL3, CCL4, IL-8, eotaxin, IP-10, MCP-1 and RANTES [63,64], suggesting that i-PRF may promote early resolution of the inflammatory process, leading to enhanced wound healing and biomaterial integration. 

FTIR and XPS are standard methods to assess protein structural changes [49]. FTIR spectra can provide information about the secondary structure of proteins that are characterized by absorption bands and that can be cataloged as amide A and B at 3500–2900 cm^−1^, as well as Amide I to VII, which appear at lower wavelengths from 1650 cm^−1^. FTIR spectra also allow the detection of the specific peptide bond vibration –CO-NH-, which represents functional groups on the protein secondary structure. In particular, the Amide I band (1600–1700 cm^−1^), which is characterized by C=O stretching vibrations, is commonly used to quantify secondary structure in proteins and polypeptides [50]. Historically, different absorption bands ranging between 1642 cm^−1^ and 1624 cm^−1^ in Amide I as well Amide A (3310–3270 cm^−1^) [51] have been linked to β-sheet conformation [52], a common motif of secondary structure in proteins. Furthermore, the adsorption of proteins on the PCL/CaP surface was also investigated via XPS by using C1s, N1s and O1s high-resolution spectra. The C1s signal deconvolutions observed in the current study clearly demonstrated similarities between the i-PRF and L-PRP spectra with components at 284.7 eV (reflective of C\C and C\N bonds), as well as 283.2 eV and 282.8 eV, which were assigned to C-C and C-H bonds, respectively. Interestingly, the 282 eV component was not present in P-PRP, which is consistent with a previous report on C1s signal deconvolution for proteins adsorbed on biomaterial surfaces [45,65]. N1s high resolution spectra demonstrated significant increases in nitrogen content once the surface was functionalized with i-PRF and showed superior binding energy at 399.6 eV and 397.6 eV, which represents O=C-N and Fe-N/Ni-N bonds, respectively. Parameters such as temperature, pH, surface topography and protein composition are crucial in the adsorption of proteins [66]. In the context of hemoderivatives, the differential protocols utilized to manufacture the fresh blood products, such as i-PRF, or anticoagulant-containing preparations, such as P-PRP, L-PRP and PLASMA, may also influence the differential structural protein conformation and corona development. 

The results of this study indicate that there was greater PCL/CaP surface coverage with i-PRF compared to the other hemoderivatives, which produced a distinct PC composition in this group. However, a limitation of the current study is the lack of identification of the proteins or group of proteins on the different experimental coronas. 

In the context of regenerative medicine approaches using hemoderivatives, the formation of the hemoderivative-derived coronas on the surface of the biomaterials may significantly influence the healing capacity. Interestingly, the methods for processing the blood products not only affected the protein content and its resulting adsorption on the polymer surface but also the morphology of the fibrin network once clotted. Indeed, the combined increase in the *g* force and the addition of CaCl_2_ (or anti-clotting reagent Sodium Citrate) on P-PRP and L-PRP resulted in thicker fibrin fibers (≈700 nm) compared to PLASMA (≈400 nm) and i-PRF (≈100 nm), as previously reported [67,68]. In contrast, i-PRF undergoes a coagulation process resembling natural clotting and is activated by the release of Factor XII, which initiates the fibrin cross-linking by converting prothrombin to thrombin, as well as fibrinogen in fibrin [69]. Therefore, this process is milder than the addition of a cross-linking agent, potentially explaining the lower diameter of the fibrin fibers, lower porosity and higher weight loss caused by a lower cross-linking percentage compared to the other groups. In turn, this may be beneficial for tissue regeneration, as a more “fragile” i-PRF fibrin mesh may facilitate cellular infiltration and the release of different growth factors and cytokines [70,71,72]. In addition, the i-PRF higher total protein adsorption, which has also been reported for various biomaterial surfaces [41,72,73], may be caused by the thinner fibrin mesh enabling an increased coverage of the PCL/CaP surface. In contrast, natural clot aims to form fibrin mesh for controlling blood loss and achieve hemostasis [74]. 

By harnessing a tailored PC, the establishment of a customized clot with less predominant β-sheet protein conformation may diminish the development of scar tissue (possibly leading to fibrous encapsulation) around implantable devices. The distinct hemoderivative-dependent PC formed on the biomaterial surface before implantation opens new opportunities for controlling the early regenerative events in the field of tissue engineering. This biomaterial surface modification can be termed “clot tissue engineering” and combines the utilization of different blood processing methods for the creation of a tailored PC capable of influencing the immune–inflammatory response and the downstream regenerative process. This strategy of pre-loading hemoderivatives on implantable devices is emerging as a promising alternative in translational medicine and could be further refined by incorporating other biological cues, autologous cells isolated from peripheral blood or inorganic compounds.

## 4. Materials and Methods

### 4.1. Scaffold Fabrication

The polycaprolactone scaffolds were fabricated by using melt electrospinning writing (MEW) in a custom-made device, as previously described [75]. Medical-grade poly (ε-caprolactone) (mPCL) (Purac ~80 kDa) pellets were placed into a 2 mL syringe and extruded at 80 °C through a blunt 21 G needle at a feed rate of 20 μL/h, by using a voltage of 7 kV and a spinneret collector distance of 15 mm. The translational velocity of the collector was set at 250 mm/min in order to obtain straight fibers, and a square wave pattern was utilized to fabricate a scaffold composed of alternating series of layers oriented at 90°, with an inter-fiber distance of 250 μm. This resulted in the fabrication of a 1 mm thick, porous melt electrospun scaffold.

### 4.2. Calcium Phosphate Coating

The coating procedure was adapted from Yang et al. [32] and involved the use of a saturated Simulated Body Fluid (SBF 10×), as previously described [42]. Briefly, the scaffolds were immersed in ethanol and placed under a vacuum for 10 min in order to remove entrapped air bubbles that could negatively impact on the coating homogeneity. The MEW scaffolds were then placed in NaOH (1 M, 37 °C. #A4782901 Gibco™, Scoresby, VIC, Australia) for 30 min. The last rinsing solution had a pH of 7, thus ensuring that early precipitation in the SBF solution would not occur. Thereafter, the SBF solution was adjusted to pH 6 with NaHCO_3_ (1M- #144-55-8, Sigma-Aldrich, Australia) under gentle stirring. This solution was filtered by using a 0.2 µm-filter (#CLS431222. Sigma-Aldrich, Australia), and 35 mL of this solution was poured into a 50 mL falcon tube containing 20 scaffolds. A 5 min vacuum treatment was then performed to ensure optimal infiltration of the solution throughout the scaffolds. The pressure was gently released to avoid early precipitation at the surface of the SBF solution. Thereafter, the samples were placed at 37 °C for 30 min, and the tubes were gently shaken every 10 min. After 30 min, the scaffolds were immersed in a fresh SBF 10× solution adjusted at pH 6 for 30 min at 37 °C. The samples were then rinsed twice in Milli Q water. A post-treatment step, consisting of immersing the scaffolds in NaOH (0.5M-#1310-73-2, Merck, Australia) at 37 °C for 30 min, was performed in order to homogenize the calcium phosphate (CaP) phase obtained after the SBF treatment, according to our published protocol [42]. Finally, the scaffolds were rinsed with distilled water 5 times and dried under vacuum until use. 

### 4.3. Morphology Characterization

The morphologies of the biphasic scaffold were investigated by using SEM. SEM was conducted by using a QUANTA200 microscope with a 10 kV acceleration voltage. The mPCL scaffolds were sputter-coated with gold for 150 s prior to visualization. Energy dispersive X-ray spectroscopy (EDX) was performed to confirm calcium and phosphate deposition. 

### 4.4. Hemoderivative Preparation

Ovine blood samples were collected from three different animals and freshly prepared in triplicate immediately after the blood was drawn, prior to coagulation. All experiments were conducted in accordance with the guidelines described by the *Australian Code of Practice for the Care and Use of Animals for Scientific Purposes* (8th edition, 2013) and received approval from the Queensland University of Technology Animal Ethics Committee (#1500001242) and ratification from The University of Queensland (QUT/156/18). All blood products were prepared by using a commercially available clinical centrifuge (Duo Centrifuge, Fixed angle rotor/radius 110 mm, Biomedent Australia) and by using various centrifugation protocols. Total PLASMA was used as biological control group. In the characterization of the blood products, 9 samples obtained from 3 different animals (*n* = 3 for each animal) were assayed. 

#### 4.4.1. Plasma Preparation

Whole blood was collected in sodium citrated tubes (#BCTCIT -Livingstone International PTY LTD. Rosebery NSW, Australia). The collected blood was manually and gently stirred to prevent coagulation and ensure homogeneity of the sample. Samples were centrifuged for 15 min at 2000× *g,* and the resulting supernatant was transferred into a fresh tube and mixed with 20 μL/mL of 10% (wt/vol) calcium chloride (#447325000-Thermo Scientific™, Australia) in Millipore water (#750023- Invitrogen™, Australia) to initiate cross-linking. Subsequently, 100 µL/scaffold was pipetted into MEW scaffolds and maintained at 37 °C until gelation. This hemoderivative volume/scaffold ratio was maintained for all subsequent experiments.

#### 4.4.2. i-PRF Preparation

For injectable platelet-rich fibrin (i-PRF), 10 mL of blood was collected in a vacuum tube (Vacuum tube i-PRF^TM^ Choukroun, Biomedent, Australia) and centrifuged at 700 rpm (60× *g*) for 3 min. Following centrifugation, the orange supernatant (≈2 mL), which represented the liquid i-PRF, was aspirated by using a syringe fitted with a 21 G needle, and 100 µL were injected in the MEW scaffold. Then, the i-PRF scaffolds were placed at 37 °C, and natural gelation occurred within 30 min. 

#### 4.4.3. Leukocyte Platelet-Rich Plasma (L-PRP) and Pure Platelet-Rich Plasma (P-PRP)

Anticoagulated preparations were performed by using previously described protocols [76,77]. Briefly, 10 mL of blood was collected by using sodium citrated tubes. The collected blood was manually and gently stirred to prevent coagulation and ensure homogeneity of the sample. 

The preparation of the L-PRP samples consisted of 2 sequential centrifugation steps; the tubes were firstly centrifuged at 2400 rpm (708× *g*) for 10 min. Thereafter, the plasma section and “buffy coat” were collected and transferred by using a micropipette to a new tube for a second centrifugation at 3600 rpm (1594× *g*) for 15 min. Most of the platelet-poor plasma (PPP) fraction was discarded, and the platelet pellet was re-suspended in a small fraction of PPP to make 2 mL of L-PRP. The gelation of the L-PRP was initiated by adding 20 μL of a 10% (wt/vol) calcium chloride in Millipore water per milliliter of L-PRP, and the 100 µL were injected in the scaffolds and maintained at 37 °C until gelation. For the P-PRP preparation, 10 mL of blood were centrifuged at 1800 rpm (398× *g*) for 8 min in order to fractionate the blood into three different layers, as previously described [40]. Thereafter, 2 mL of superficial platelet-poor plasma (PPP) was discarded, and 2 mL of platelet-rich plasma was collected with a micropipette, with care taken to avoid turbulence and prevent buffy coat aspiration. Similarly to the L-PRP preparation, gelation was performed by adding 20 μL of a 10% (wt/vol) calcium chloride in Millipore water per mL of P-PRP, and 100 µL were added in the scaffolds.

### 4.5. Blood Product SEM Characterization

The different blood-product-loaded scaffolds were characterized by SEM. The resulting samples were transferred into 6-well plates (#150239, Thermo Scientific™, Australia) and fixed with 2.5% glutaraldehyde (#A17876.AP, Thermo Scientific™, Australia) in PBS (#20012027, Gibco™, Australia) at pH 7.4 for 2 h and frozen at −80 °C for 2 h. The samples were then freeze-dried (FreeZone Plus 2.5, Kansas City, MO, USA) at −80 °C/0.006 mbar for 24 h and finally gold-coated for visualization by using a QUANTA200 microscope, as described above. SEM micrographs were analyzed by using the open-source software ImageJ (ImageJ, US National Institutes of Health, Bethesda, MA, USA) for fibrin fiber diameter, porosity and pore size. To this end, three technical replicates from three biological replicates were used for characterization (*n* = 9). For fibrin fiber diameter, three representative images per sample were directly measured. For pore size and porosity, three representative images per sample were uploaded on ImageJ and converted to 8-bit binary images. Image thresholding was automatically applied, and the ND (nearest distance) plugin was run to calculate the average pore size. Similarly, porosity was calculated as previously reported [78] by using the JPOR plugin (ImageJ). Values were presented as mean and standard deviation.

### 4.6. Cross-Linking Assay

A Ninhydrin assay was conducted to quantify the degree of immobilization (cross-linking) [79] and was calculated as the ratio between consumed and free amino groups in two technical replicates from three biological replicates (*n* = 6). The samples (100 µL) were first frozen at −80 °C for 2 h and subsequently freeze-dried for 24 h. For analysis, the samples were weighted and placed into Eppendorf tubes (#339650, Thermo Scientific™, Australia) and resuspended with 2 mL of 2% (*w*/*v*) Ninhydrin (#A10409.09, Thermo Scientific™, Australia) for 15 min at 100 °C. The solution was then allowed to cool at RT, and 1.5 mL of 50% ethanol was added. 

A standard curve was prepared by using glycine (1–0.031 mM #15527013, Thermo Scientific™, Australia), and the absorbance at 570 nm was determined by using a spectrophotometer (POLARstar Omega, Offenburg, Germany). 

The concentration of free NH_2_ groups was calculated by using the following formula:

(1) $$Free\;NH2\;group\;=\frac{\left(\mathrm{Free}\;\mathrm{aminoacid}\;\left[\;\right]\right)\times \left(\mathrm{NH}2\;\mathrm{molecular}\;\mathrm{weight}\right)}{Sample\;weight}$$

where concentration was determined from the standard curve of glycine concentration vs. absorbance, and NH_2_ molecular weight was calculated as the sum of the atomic weight values (16.022 g) divided by sample weight (g).

Subsequently, the degree of cross-linking was calculated with free NH_2_ groups of non-cross linked and cross-linked samples by using the formula [80]:

(2) $$Degree\;of\;Cross\text{-}linking\;=\frac{\left(Free\;NH2\;non\text{-}cross\text{-}linked\right)-\left(Free\;NH2\;crosslinked\right)}{Free\;NH2\;non\text{-}cross\text{-}linked}\times 100$$

### 4.7. Weight Loss

The blood products (*n* = 3 for each type) were weighed and subsequently placed in a 6-well plate containing 2 mL of 0.01% trypsin (#15090046- Gibco™, Australia) and incubated at 37 °C for 3 days with a daily replacement of the trypsin solution. Weight loss was normalized at the initial weight of the hemoderivative/scaffold assembly and expressed as percentage of remaining weight.

### 4.8. pH

In order to determine pH values, the hemoderivatives (i-PRF, L-PRP, P-PRP and PLASMA) samples (2 mL) were transferred to 6-well plates in 5 mL of α-MEM (#12561056-Thermo Scientific™, Australia) supplemented with 1% Penicillin–Streptomycin (#15140122- Gibco™, Australia) and placed on a shaker (WYC-290A, Labwit Scientific Pty Ltd., Ashwood, VIC, Australia) in the incubator at 5% CO_2_ atmosphere and at 37 °C. After 1, 3, 7 and 10 days, the pH was measured by using a pH meter (510 Eutech Instruments, Singapore) at 37 °C. In this experiment, an additional control group consisting of 5 mL of α-MEM media with 1% PS was prepared with a pH 7.35, which mimicked the physiological pH of blood. For each group, three different samples at each time point were utilized.

### 4.9. Quantification of Protein Absorption from the Hemoderivatives

The protein adsorption on both CaP-coated and pristine PCL scaffolds was determined by soaking the scaffolds in PBS (pH 7.4- #10010023-Thermo Scientific™, Australia) overnight at 37 °C. Thereafter, the scaffolds were transferred into a plate containing 2 mL of un-cross-linked i-PRF, P-PRP and L-PRP at various concentrations (1%, 2.5%, 5%, 10%, 20% and 50%) for 60 min by using PBS as a diluent. The scaffolds were washed three times with PBS for 10 min to remove the soft PC (non-specifically adsorbed proteins). The scaffolds were then transferred into a plate containing 200 μL of ice-cold lysis buffer (2% Sodium Dodecyl Sulfate: #28312, Thermo Fisher Scientific Inc., Australia) supplemented with a protease inhibitor cocktail (P8340, Merck, Bayswater, VIC, Australia), as previously reported [81]. The samples were placed on ice under a shaking incubator at 800 rpm for 3 h to collect the adhered proteins constituting the so-called “hard corona” [82]. The supernatant was collected and evaluated by using a BCA protein assay reagent (Novagen TB380, Sigma-Aldrich Pty Ltd., Australia). Two technical replicates from three biological replicates were used for characterization (*n* = 6) per concentration per hemoderivative.

### 4.10. Protein Absorption Spectroscopy (FTIR-XPS)

Protein surface adsorption was analyzed via spectroscopy by using Fourier-transform infrared spectroscopy (FTIR) and X-ray photoelectron spectroscopy (XPS). The scaffolds were immersed in PBS overnight at 37 °C and subsequently incubated in a 100 µL solution of the different hemoderivatives (100%) for 60 min. Three biological replicates per hemoderivative were assayed by using two technical replicates (*n* = 6 for each hemoderivative). The samples were then transferred to a plate and placed in a −80 °C freezer for 1 h prior to freeze-drying for 24 h. An FTIR spectroscope (Nicolet 5700, Thermo Fisher Scientific Waltham, MA, USA) was used to verify the presence of the different blood fractions on the PCL scaffolds. The spectra were acquired via a collection of 64 scans over a scan range between 4000 cm^−1^ and 400 cm^−1^ at a resolution of 8 cm^−1^. XPS data were acquired by using a Kratos Axis ULTRA spectrometer (Kratos Analytical Ltd., Manchester, UK) incorporating a 165 nm hemispherical electron energy analyzer. The incident radiation was monochromated Al Kα X-rays (1486.6 eV) at 150 kV and 0.1 mA (150 W). Survey scans were taken at a pass energy of 160 eV, over a binding energy range of 1200–0 eV and a dwell time of 100 ms. Narrow scans were taken at a pass energy of 20 eV, with 0.05 eV steps and a dwell time of 250 ms. The analysis chamber had a base pressure of 1.0 × 10^−9^ torr, and the pressure during the analysis was 1.0 × 10^−8^ torr. High-resolution spectra were resolved into individual Gaussian–Lorentzian peaks by using CasaXPS software (v 2.3.17-Clearwater, FL, USA) to calculate atomic concentrations and carry out peak fitting to the narrow scan data. Component energies, number of peaks and peak widths (full width at half maximum (fwhm) were fixed initially), and refinement was performed for peak heights only. 

### 4.11. Protein Dynamic Binding

Protein dynamic binding from the various hemoderivatives (i.e., i-PRF, P-PRP, L-PRP and PLASMA) was analyzed on PCL/CaP scaffolds. Here again, the scaffolds were incubated in PBS overnight at 37 °C and subsequently transferred into a plate containing 200 µL of 1 of the various hemoderivatives without dilution (100%) for 1 min, 5 min, 15 min, 30 min and 60 min at 37 °C. Two technical replicates from three biological replicates (*n* = 6) per time point per hemoderivative were used for this experiment. The scaffolds were washed three times with PBS for 10 min to remove the soft PC and transferred into a plate containing 200 μL of ice-cold lysis buffer supplement with a protease inhibitor cocktail and maintained on ice under shaking incubation at 800 rpm for 3 h to collect the adhered proteins. Samples were reduced by adding β-mercaptoethanol (6 µL per 94 µL of protein solution. #21985023- Gibco™, Australia), incubated for 5 min, vortexed, transferred onto a hot block (#1199A66 Digital Heating Shaking Dry Bath, Thomas Scientific, NJ, USA) at 100 °C for 5 min and then cooled to RT. The resultant supernatant was re-suspended (1:1 vol) in sample buffer (#1610747, Bio-rad, Australia) and loaded onto 4–12% Bis-Tris SDS-PAGE gels (NuPAGE Bis-Tris protein gels, Thermo Fisher Scientific Inc., Australia). All the gels were ran in MOPS buffer (NuPAGE MOPS SDS Running Buffer, Thermo Fisher Scientific Inc., Australia) at 200 V for 50 min. The presence of protein components was visualized via Coomassie staining (LC6065 SimplyBlue™ SafeStain, Thermo Fisher Scientific Inc., Australia). Gels were immediately scanned (Chemidoc MP Imager, Bio-Rad Laboratories, Australia), maintaining the automatic optimized exposure time for intensity bands setting. Captured images were analyzed by using the Analyzing Electrophoretic Gel plugin in ImageJ, which was previously calibrated for optical density. Line profile plots were generated, and areas of the peaks were calculated accordingly. Data were subsequently expressed in percentage (%).

### 4.12. Statistical Analysis

Statistical analysis was conducted via one-way ANOVA, with a Bonferroni post hoc test, by using GraphPad Software v. 7 (GraphPad Software, La Jolla, CA, USA). Statistical significance was considered to be at *p* < 0.05. All data were expressed as mean ± standard deviation. 

## 5. Conclusions

The properties of the protein corona on PCL surfaces are significantly influenced by different hemoderivatives in a highly specific manner. i-PRF demonstrated a predominant adsorption of low-to-medium molecular weight proteins after 60 min, which was devoid of higher MW proteins. Anticoagulated protocols (L-PRP, P-PRP) demonstrated high similarities in their respective protein layers, which were remarkably different in their composition and conformation to i-PRF and PLASMA. The characteristics of the PC may positively influence the immune–inflammatory response and benefit the regenerative outcome in patients receiving implantable devices. The composition and structural properties of hemoderivatives can be used to tailor the protein coating of biomedical devices before surgical implantation in a bench-to-bedside manner. Autologous blood products rich in fibrin used as a biological coating in biomaterials might contribute to the reduction of scar formation and promote bio-integration (i.e., fibro-integration; osseointegration). Further investigation of hemoderivative functionalized tissue-engineering scaffolds is required to elucidate the relationship between the different PCs and the resultant downstream immune response and regenerative capacity. 

## Figures and Tables

**Table 1:** Summary of the C, O and N proportions as obtained by XPS.

Sample	C 1s (%)	O 1s (%)	N 1s (%)	O/C	N/C	NH/NH_2_ (%)
i-PRF	59.6	25.5	4.3	0.43	0.075	1.7
P-PRP	60.2	25.03	2.6	0.41	0.043	2.2
L-PRP	66.7	19.5	5.6	0.29	0.083	5.2
PLASMA	55.0	22.6	10.3	0.42	0.19	2.8

## Data Availability

The data presented in this study are available on reasonable request from the corresponding author.

## Supplementary Materials

The supporting information can be downloaded at: https://www.mdpi.com/article/10.3390/ijms24076162/s1.
