# Supplementary material for: Characterization of the Protein Corona of Three Chairside Hemoderivatives on Melt Electrowritten Polycaprolactone Scaffolds
Source: Int J Mol Sci. 2023 Mar 24;24(7):6162. doi: 10.3390/ijms24076162 (PMC10094244; doi:10.3390/ijms24076162)
Supplement: Supplementary file 1 [file ijms-24-06162-s001.zip › ijms-2242867-supplementary.pdf]

Supplementary material

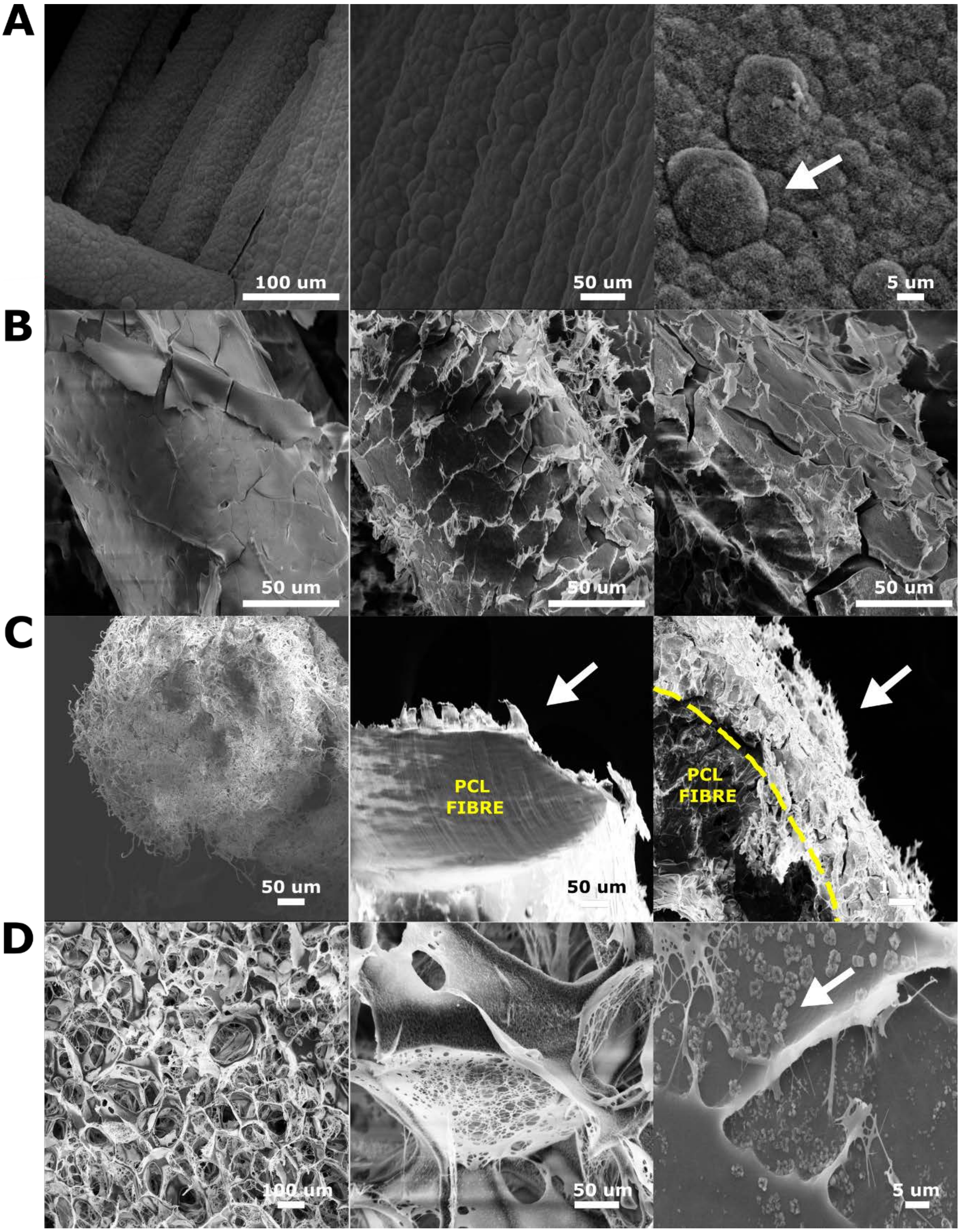

SM Figure S1. Rows from left to right: (**A**) PCL/CaP scaffolds at low, middle and high magnification showing the characteristic CaP nodules deposition on surface (white arrow). (**B**) PCL/CaP scaffolds fibres covered with i-PRF, P-PRP and L-PRP respectively. PCL surface is visible under isolated fractures on surfaces due to of sample processing (freeze dry). (**C**) Cross section of PCL fibres surrounded by fibrin network (white arrows) from hemoderivatives. (**D**) Hemoderivative fibrin network between PCL fibres have shown macro (left) and micro porosity (middle) along with Platelets (right) can be seen of the surface (white arrow) specially in close contact with fibrin mesh.
